# Association between Anxiety and Depression with Sleep Quality among Iraqi Polypharmacy Older Adults: A Cross-Sectional Study

**DOI:** 10.1192/j.eurpsy.2025.2323

**Published:** 2025-08-26

**Authors:** G. Alhashem, M. Aldawoudi, M. AlZayadi

**Affiliations:** 1 Pharmacy, AlSafwa University College, Karbala, Iraq

## Abstract

**Introduction:**

Many definitions have been proposed for polypharmacy, but the most common is the concurrent use of five or more medications. It is a growing concern among older adults and is associated with numerous adverse effects and drug-drug interactions. Beyond its impact on physical health, research suggests that polypharmacy may also affect mental health, which could potentially be linked to poor sleep quality.

**Objectives:**

The current study objective is to investigate the sleep quality of polypharmacy older adults and its association with depression and anxiety in this population.

**Methods:**

A cross-sectional study was conducted in Iraq from June to August 2024. A total of 245 participants were selected from internal medicine wards in hospitals and private clinics. All participants were aged 50 years or older and had been taking five or more medications for at least 90 days. The study questionnaire comprised three main sections: demographic data, the Patient Health Questionnaire-4 (PHQ-4), and the Pittsburgh Sleep Quality Index (PSQI). A PSQI score greater than 5 was used to identify poor sleepers. The association between poor sleep and other factors was assessed by using chi-squared tests and binary logistic regression.

**Results:**

The sample consisted of 245 polypharmacy patients, with a mean age of 61.5 ± 12.4 years. Of the participants, 150 (61%) were female and 95 (39%) were male. Anxiety was observed in 95 (38.8%) participants, depression in 96 (39.2%), and poor sleep quality in 189 (77.1%). The study found significant associations between both anxiety (OR = 3.4 [95% CI: 1.55-7.57], p = 0.002)and depressive symptoms (OR = 2.43 [95% CI: 1.15-5.15], p = 0.020) with poor sleep quality.

**Conclusions:**

Our study suggests that Iraqi polypharmacy older adults suffer from poor sleep quality, with depression and anxiety potentially exacerbating this issue. The findings suggest that mental health support is necessary for older adults with polypharmacy.

**Disclosure of Interest:**

None Declared

